# Less gap imbalance with restricted kinematic alignment than with mechanically aligned total knee arthroplasty: simulations on 3-D bone models created from CT-scans

**DOI:** 10.1080/17453674.2019.1675126

**Published:** 2019-10-15

**Authors:** William Blakeney, Yann Beaulieu, Marc-Olivier Kiss, Charles Rivière, Pascal-André Vendittoli

**Affiliations:** a Department of Surgery, CIUSSS-de-L’Est-de-L’Ile-de-Montréal, Hôpital Maisonneuve Rosemont, Montréal, Québec, Canada;; b Department of Surgery, Albany Health Campus, Albany, Australia;; c Department of Surgery, Université de Montréal, Montréal, Québec, Canada;; d Adult Reconstruction and Joint Replacement, South West London Elective Orthopaedic Centre, MSK-Lab—Imperial College London, London, UK

## Abstract

Background and purpose — Mechanical alignment techniques for total knee arthroplasty (TKA) introduce significant anatomic alteration and secondary ligament imbalances. We propose a restricted kinematic alignment (rKA) protocol to minimize these issues and improve TKA clinical outcomes.

Patients and methods — rKA tibial and femoral bone resections were simulated on 1,000 knee CT scans from a database of patients undergoing TKA. rKA was defined by the following criteria: independent tibial and femoral cuts within 5° of the bone neutral mechanical axis, with a resulting HKA within 3° of neutral. Imbalances in the extension space, flexion space at 90°, medial compartment and lateral compartment were calculated and compared with measured resection mechanical alignment (MA) results. 2 MA techniques were simulated for rotation using the surgical transepicondylar axis (TEA) and 3° to the posterior condyles (PC).

Results — Extension space imbalances ≥ 3 mm occurred in 33% of TKAs with MA technique versus 8.3% with rKA (p < 0.001). Similarly, more frequent flexion space imbalance ≥ 3mm was created by MA technique (TEA 34% or 3° PC 15%) versus rKA (6.4%, p < 0.001). Using MA with TEA or PC, there were only 49% and 63% of the knees respectively with < 3 mm of imbalance throughout the extension and flexion spaces and medial and lateral compartments versus 92% using rKA (p < 0.001).

Interpretation — significantly fewer imbalances are created using rKA versus MA for TKA. rKA may be the best compromise, by helping the surgeon to preserve native knee ligament balance during TKA and avoid residual instability, whilst keeping the lower limb alignment within a safe range.

Human lower limb anatomy varies widely, and pathological changes increase this variability further (Almaawi et al. 2017, Hirschmann et al. 2019b, Moser et al. 2019). A standardized, systematic approach, using right-angled femoral and tibial bone cuts (Mechanical Alignment) with the concept of parallel and equal flexion and extension gaps, was introduced early in the development of TKA (Freeman et al. 1973, Scuderi et al. 2001). As very few individuals have neutral femoral and tibial mechanical axes (0.1% of a population of 4,884 patients scheduled for TKA), MA leads to important anatomic alterations for many subjects (Bellemans et al. 2012, Almaawi et al. 2017). This results in unequal bone resections with resultant imbalances (Blakeney et al. 2019a). Multiple ligament release techniques and algorithms have been proposed to re-balance the joint gaps. This resulted in many surgeons thinking of TKA as a soft-tissue surgery to balance the gap modification linked to these standardized bone-cut orientations (Whiteside 2002). There is, however, debate as to whether the knee’s collateral ligament laxities are modified in knees with less than 15° of deformity (McAuliffe et al. 2017, 2019). Soft-tissue releases are technically demanding, unpredictable, and can even introduce further imbalance (Kumar and Dorr 1997). Extensive releases may change the position of the joint line (Yoshii et al. 1991), which may have an adverse effect on the knee’s range of movement or the function of the extensor mechanism (Walker and Garg 1991) and worsen the clinical outcome (Martin and Whiteside 1990, Unitt et al. 2008). TKA joint gap imbalance has been associated with abnormal kinematics, decreased range of motion, condylar lift-off, loosening, wear and is a frequent cause of revision surgery, with rates varying from 21% to 35% (Wasielewski et al. 1994, Dennis et al. 2010, Gustke et al. 2014, Le et al. 2014).

The restoration and preservation of pre-arthritic knee anatomy and ligament laxities during TKA has gained interest in recent years (Hirschmann et al. 2019a). The kinematic alignment (KA) technique represents a resurfacing of articular surfaces, removing equivalent amounts of bone and cartilage to match implant thickness (Howell et al. 2013). Concerns remain about restoring extreme anatomies, which may not be compatible with current TKA prostheses and fixation methods. Some knee anatomies may be inherently biomechanically inferior, or may have been altered by trauma, tumors, childhood deformity, or previous surgery. Keeping in mind these uncertainties, the senior author (PAV) developed a restricted KA (rKA) protocol (Hutt et al. 2016). rKA aims to perform KA bone resections for most cases, but performing adjustments for patients outside a “safe range” defined by the following criteria: independent tibial and femoral cuts must be within 5° of the mechanical axis of the respective bone and the overall resulting Hip–Knee–Ankle angle (HKA) must fall within 3° of neutral. Using a database of 4,884 CT scans of lower limbs for patients scheduled for TKA, a previous study demonstrated that in half of osteoarthritic knees there was no difference from standard KA resections with the rKA protocol, and mean anatomical corrections of 0.5° for medial proximal tibial angle (MPTA) and 0.3° for lateral distal femoral angle (LDFA) were needed to fit 4,062 cases (83%) (Almaawi et al. 2017).

The objective of this study was to calculate bone resection thicknesses and resulting imbalances in the flexion/extension spaces and medial/lateral compartments, by simulating rKA protocol on 3-D bone models created from 1,000 CT scans of patients undergoing TKA and to compare the imbalances with previously published measured resection mechanical alignment (MA) results (Blakeney et al. 2019a). The study hypothesis was that the rKA protocol would reduce imbalance in the extension and flexion spaces and in the medial and lateral compartments versus MA.

## Material and methods

The data from this study were taken from a database of 1,000 consecutive lower limb CT scans, on patients scheduled for TKA using patient-specific instrumentation (PSI) with the MyKnee system (Medacta International, Switzerland). Mean HKA from the supine CT scan was 177° (SD 5.0, range 164–194). There were 730 (73%) varus cases and 270 (27%) valgus cases. We then calculated a computed HKA, which was the sum of LDFA and MPTA.

Tibial and femoral bone resections were simulated according to our rKA protocol. The “safe range’’ is defined by the following criteria: independent tibial and femoral cuts within 5° of the bone’s neutral mechanical axis and a resulting HKA within 3° of neutral. The algorithm was applied in 2 steps. For knee anatomy that fell outside the proposed safe range, the LDFA and MPTA were corrected independently by setting them to closest value: ±5° from neutral. After the independent femoral or tibial corrections, if HKA remained > 3° of varus or valgus (aiming to maintain the femoral flexion axis as closely as possible) we adjusted the MPTA to bring the HKA within the safe range of ±3°.

The distal femoral and proximal tibial cut resections were set at 8 mm from the distal femoral condyles and 8 mm from the proximal tibial plateaus. If corrections to the MPTA or LDFA were required per above protocol, 1 resection was maintained at 8 mm and the other reduced accordingly. An 8 mm resection thickness was based on an implant size of 10 mm (bone +2 mm of cartilage) (Li et al. 2005). Equal medial and lateral posterior femoral resections of 8 mm thickness were simulated on all scans (no femoral rotation).

After simulation of the bone cuts, the gap sizes were calculated as the sum of the femoral and tibial bone resections. Using a CT scan without the cartilage, the target bone resection was 16 mm (+ 2 x 2 mm for cartilage thicknesses corresponds to a total implant thickness of 20 mm). 4 gaps were measured: medial and lateral gaps in both extension and 90° of flexion. An “imbalance” was defined as the difference between 2 gaps. These imbalances are created when the protocol modifies the KA resection to stay within the safe range. A clinically important imbalance was considered to be 3 mm or greater.

4 imbalances were calculated:

- extension space: medial gap in extension—the lateral gap in extension;
- flexion space: medial gap in flexion—the lateral gap in flexion;
- medial compartment: medial gap in extension—medial gap in flexion;
- lateral compartment: lateral gap in extension—lateral gap in flexion.

The mean was also calculated based on absolute values.

We compared these results with previously reported results for MA technique using the same database of patients (Blakeney et al. 2019a). MA femoral rotation was assessed with 2 techniques: femur aligned with the surgical transepicondylar axis (TEA) or aligned with 3° of external rotation to the posterior condyles (PC). A resection plane, aligned with the posterior condyles (8 mm thickness medially and laterally) was rotated to the appropriate angle (TEA or PC) using a central pivot.

### Statistics

Descriptive statistics were calculated to summarize patient anatomy and resection measures. To compare continuous variables between rKA and MA techniques, 2-sample t-tests for independent groups were used. Paired t-tests were used to compare continuous variables between PC and TEA techniques. All tests were 2-tailed, with a significance level of p < 0.001 (to allow for multiple comparisons). Chi-squared or McNemar tests were used to compare categorical data.

### Ethics, funding, and potential conflicts of interest

This article used anonymous data from an existing collection of CT scans and does not contain any studies with human participants performed by any of the authors. Informed consent for this type of study is not required. Funding was received from OMeGA Medical Grants Association fellowship support. The authors have no potential conflict of interest.

## Results

### Lower limb alignment

**Figure 1.**
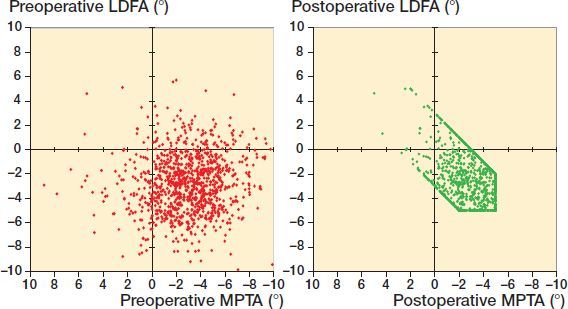
LDFA and MPTA comparing preoperative and postoperative distributions.

Table 1 presents the preoperative lower limb alignment and the resulting effects of the rKA protocol. With computed HKA (LDFA + MPTA), there were 521 (52%) varus and 479 (48%) valgus cases preoperatively versus 505 (51%) and 495 (49%) after rKA. Although there was no significant mean difference in HKA after rKA, rKA significantly modified the LDFA and MPTA compared with preoperative values (p < 0.001). With rKA, LDFA and MPTA were independently modified for 18% and 45% of cases respectively. The femoral valgus and tibial varus were reduced by a mean of 0.4° for both (absolute modification for femur 0.4° and tibia 1.6°). Modifications of both the LDFA and MPTA were needed in 10% of cases. Figure 1 shows the native LDFA and MPTA versus the resulting cut orientations after rKA protocol application.

**Table 1.** Lower limb alignment of pre-operative anatomy compared with after rKA. Values are mean (SD) [range] degrees.

	Preoperative anatomy	After rKA	p-value	rKA angle modification	
				whole cohort	cases modified
HKA angle absolute values	180 (3.6) [168 to 191]	180 (2.2) [177 to 183]	0.6	0.0 (1.8) [–8.3 to 8.8] 1.0 (1.5) [0.0 to 8.8]	–0.1 (2.5) [–8.3 to 8.8] 1.9 (1.7) [0.0 to 8.8)
LDFA absolute values	–2.8 (2.4) [–9.8 to 5.8)	–2.6 (2.1) [–5.0 to 5.0]	< 0.001	0.2 (0.6) [–0.8 to 4.8] 0.2 (0.6) [0.0 to 4.8]	0.4 (0.8) [–0.8 to 4.8] 0.4 (0.8) [0.0 to 4.8]
MPTA absolute values	2.9 (2.6) [–8.9 to 9.9]	2.7 (1.7) [–5.0 to 5.0]	< 0.001	–0.2 (1.6) [–8.3 to 8.8] 0.8 (1.4) [0.0 to 8.8]	–0.4 (2.2) [–8.3 to 8.8) 1.6 (1.7) [0.0 to 8.8]
TEA angle	5.2 (1.8) [0.3 to 9.7)				

HKA angle: hip-knee-ankle angle (computed as LDFA + MPTA).

LDFA: lateral distal femoral angle.

MPTA: medial proximal tibial angle.

TEA angle: degrees of external rotation of the transepicondylar axis to the posterior condyles.

rKA angle modification: rKA minus native anatomy.

### Extension space

**Figure 2.**
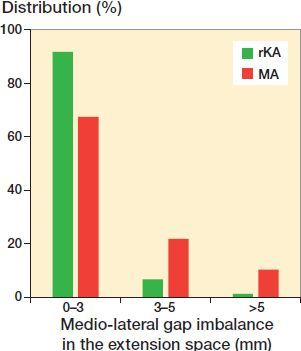
Distribution of medio-lateral gap imbalance in the extension space for rKA and MA techniques (p < 0.001).

With rKA, the created gaps in the medial and lateral compartments were maintained within 2 mm (14–16 mm) for 94% of cases, compared with 50% and 48% with MA (McNemar test p < 0.001 and p < 0.001). In other cases, the gaps were reduced (Tables 2 and 3). The mean extension space imbalance was 0.8 mm with rKA and 2.4 mm for MA (p < 0.001, Table 3). There were significantly fewer cases having imbalance ≥ 3mm with rKA (8.3%) vs. MA (33%), and ≥ 5mm with rKA (1.5%) vs. MA (11%) (p < 0.001, Figure 2).

**Table 2.** Distribution of medial and lateral gap sizes in the extension space for MA and rKA techniques. Values are percentages

Extension space (mm)	Medial extension gap		Lateral extension gap	
	MA	rKA	MA	rKA
< 12	17	1.5	17	1.1
12–13	33	5.0	35	5.0
14–15	35	15	32	14
16	15	79	15	80
p-value	< 0.001		< 0.001	

The gap size in extension is the sum of the distal femoral bone resection and tibial bone resection.

Note: The aim is for a resection of 16 mm.

**Table 3.** Medial and lateral gaps modification in the extension space and resulting medio-lateral difference in mm for MA and rKA techniques. Values are mean (SD) [range]

	MA	rKA	p-value
Medial gap	–2.7 (1.9) [–8.9 to 0.0]	–0.4 (1.0) [–6.5 to 0.0]	< 0.001
Lateral gap	–2.7 (1.9) [–9.5 to 0.0]	–0.4 (1.0) [–7.1 to 0.0]	< 0.001
ΔML	0.0 (3.0) [–9.5 to 8.9]	0.0 (1.5) [–7.1 to 6.5]	0.7
absolute values	2.4 (1.9) [0.0 to 9.5]	0.8 (1.3) [0.0 to 7.1]	< 0.001

The gap size modification is the sum of the distal femoral bone resection and tibial bone resection minus 16 mm (resection goal).

ΔML: lateral gap minus medial gap; a negative value in the row represents a greater medial space than lateral space, whereas a positive value represents a greater lateral than medial space.

### Flexion space at 90°

**Figure 3.**
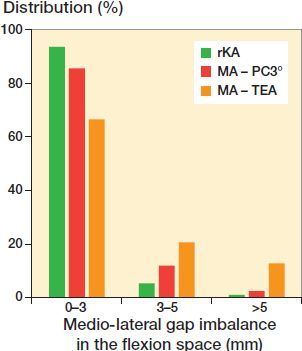
Distribution of medio-lateral gap imbalance in the flexion space for rKA and MA with PC 3° (p < 0.001) or TEA (p < 0.001) techniques.

Mean created gaps were reduced significantly more with MA PC vs. rKA (medial and lateral, p < 0.001) and with MA TEA only in the lateral compartment (p < 0.001, Table 4). Using rKA, mean flexion space imbalance was 0.7 mm versus 1.6 mm for MA PC (p = 0.001) and 2.6 mm for MA TEA (p < 0.001). There were significantly fewer cases having imbalance ≥ 3mm with rKA (6.4%) vs. MA PC (15%) and MA TEA (34%), and imbalances ≥ 5 mm for rKA (1.1%) vs. MA PC (2.5%) or MA TEA (13%) (p < 0.001, Figure 3).

**Table 4.** Medial and lateral gaps modification in the flexion space and resulting medio-lateral difference in mm for MA PC method, MA TEA method, and rKA techniques. Values are mean (SD) [range]

	MA PC method	MA TEA method	rKA	p-value: rKA vs	
				MA PC	MA TEA
Medial gap	–1.3 (1.8) [–6.5 to 1.7]	–0.4 (1.9) [–6.8 to 4.3]	–0.4 (1.0) [–6.5 to 0.0]	< 0.001	0.07
Lateral gap	–1.4 (0.6) [–7.3 to –0.9]	–2.4 (1.0) [–7.2 to –0.1]	–0.2 (0.8) [–6.0 to 0.0]	< 0.001	< 0.001
ΔML	–0.1 (2.1) [–8.4 to 5.4]	–2.0 (2.6) [–10 to 5.7]	0.2 (1.4) [–6.1 to 6.5]	< 0.001	< 0.001
absolute values	1.6 (1.3) [0.0 to 8.4]	2.6 (2.0) [0.0 to 10]	0.7 (1.2) [0.0 to 6.5]	< 0.001	< 0.001

ΔML, see Table 3.

### Medial and lateral compartment imbalances

With rKA, mean medial compartment imbalance was 0 mm vs. 1.4 mm with MA PC (p = 0.001) and 2.4 mm with MA TEA (p < 0.001) (Table 5). Mean lateral compartment imbalance was 0.2 mm with rKA vs. 1.8 mm with MA PC (p < 0.001) and 1.6 mm with MA TEA (p = 0.001). In 4.4% of rKA vs. 16% of MA PC and 33% MA TEA, there was a mismatch between flexion and extension gaps, with an extension gap too small and a flexion gap too large or vice versa (Table 6). This means, for example, that releasing a tight extension gap may increase an already loose flexion gap.

**Table 5.** Flexion–extension gap differences (ΔFE) in mm for the medial and lateral compartments for MA PC method, MA TEA method, and rKA techniques. Values are mean (SD) [range]

	MA PC method	MA TEA method	rKA	p-value: rKA vs	
				MA PC	MA TEA
Medial ΔFE	–1.4 (0.6) [0.9 to 6.6]	–2.4 (1.0) [–8.2 to –0.3]	0.0 (0.0) [–0.6 to 0.0]	< 0.001	<0 .001
absolute values	1.4 (0.6) [–6.6 to –0.9]	2.4 (1.0) [–8.2 to –0.3]	0.0 (0.0) [0.0 to 0.6]	< 0.001	< 0.001
Lateral ΔFE	–1.3 (1.8) [–6.5 to 1.7]	–0.3 (1.5) [–6.7 to 4.4]	–0.2 (0.5) [–3.7 to 0.0]	< 0.001	0.005
absolute values	1.8 (1.3) [0.0 to 6.5]	1.6 (1.1) [0.0 to 6.7]	0.2 (0.5) [0.0 to 3.7]	< 0.001	< 0.001

ΔFE: extension gap minus flexion gap; a negative value represents a greater flexion space than extension space, whereas a positive value represents a larger extension than flexion space.

**Table 6.** Percentage of knees with medial or lateral flexion-extension gap mismatch for MA PC method, MA TEA method, and rKA techniques

	Medial compartment			p-value: rKA vs		Lateral compartment			p-value: rKA vs	
	MA PC	MA TEA	rKA	MA PC	MA TEA	MA PC	MA TEA	rKA	MA PC	MA TEA
Ext. gap < 15 mm and flex. gap ≥ 16 mm	5.2	23	0	< 0.001	< 0.001	0	0	4.4	< 0.001	< 0.001
Ext. gap ≥ 16 mm and flex. gap < 15 mm	0	0	0	N/A	N/A	10	9.8	0	< 0.001	< 0.001
Total	5.2	23	0	< 0.001	< 0.001	10	9.8	4.4	< 0.001	< 0.001

### Combined imbalances

With rKA, the percentage of knees with space imbalances < 3mm in both extension and flexion was 92% vs. 63% with MA PC (p < 0.001) and 49% with MA TEA (p < 0.001) (Table 7).

**Table 7.** Percentage of knees where the medio-lateral gap mismatch is present in both the extension and flexion spaces for MA PC method, MA TEA method and rKA techniques

				p-value: rKA vs	
Gap mismatch	MA PC	MA TEA	rKA	MA PC	MA TEA
≤ 3 mm	63	49	92	< 0.001	< 0.001
≤ 5 mm	89	81	99	< 0.001	< 0.001
> 5 mm	1.9	3.8	1.1	0.1	< 0.001

NB: Data analyzed for varus and valgus native HKA separately can be found in the Supplementary data.

## Discussion

This study demonstrated that rKA produced less imbalance than an MA technique for TKA. rKA significantly reduced the cases with imbalance ≥ 3mm created by MA technique in the extension and flexion spaces, and in the medial and lateral compartments.

The extension space is created by the distal femoral and proximal tibial cut orientations and resection levels. The MA technique, using the most prominent joint surface as a reference for resection thickness (mostly the medial femoral condyle and lateral tibial plateau), will intrinsically tend to reduce the extension medial gap in varus knees and lateral gap in valgus knees (Blakeney et al. 2019a). In contrast, the KA technique aims to restore the pre-arthritic joint surface orientations by removing corresponding bone thickness to the implant thickness, thus re-creating native joint gaps. Using the rKA protocol, gaps will be modified in cases where the patient’s anatomy falls outside our safe range, requiring adjustments to be performed. In this study, rKA maintained the extension gaps within 14–16 mm (16 mm meaning no gap modification) for 94% on the medial compartment vs. 50% with MA (Table 2). On the lateral side, it was 94% with rKA vs. 48% with MA. Extension space balance was also significantly improved with rKA, where only 8.3% had an imbalance ≥ 3 mm vs. 33% with MA (Figure 2). This means that frequency and magnitude of soft-tissue release to balance the extension space would be significantly reduced with rKA TKA compared with MA.

The flexion space is created by the tibial and posterior femoral cut orientations and resection levels. To obtain a balanced flexion space with MA, femoral external rotation should match the tibial cut orientation. With MA, using a 90° cut on the tibial side reduces its anatomic varus by a mean of 3° (Bellemans et al. 2012). This is why using MA with PC 3° was shown to result in fewer flexion space imbalances versus TEA where the mean external rotation was 5° (Blakeney et al. 2019a). Since valgus knees frequently have tibial mechanical axes near neutral or in valgus (Alghamdi et al. 2014, Almaawi et al. 2017), increasing femoral external rotation resulted in even greater imbalance. With KA, bone resection equivalent to the implant thickness will be removed from the proximal tibia and the posterior condyles (neutral femoral rotation parallel to PC), thus maintaining joint flexion gaps. With our rKA protocol, gaps will be modified in cases where the patient’s MPTA fell outside the safe range of ±5°. We found that rKA significantly reduced flexion space imbalances in comparison with MA. In cases where an MPTA tibial adjustment is needed (generally reducing the varus), one could ask if we should apply some femoral external rotation accordingly to balance the flexion space. The senior author abandoned this practice early on with rKA, trying to favor maintenance of a femoral component aligned as closely as possible with the femoral flexion (cylindrical) axis (Eckhoff et al. 2007).

In addition to medio-lateral space equilibration, flexion–extension gap symmetry is considered to be a goal in TKA. Our results show that the rKA technique creates significantly fewer medial and lateral compartment imbalances versus MA (Table 5). Moreover, using MA, there were a high number of cases with an overly tight flexion gap with an overly loose extension gap, or vice versa (Table 6). Attempting to correct the tightness of these knees in one position is likely to worsen the laxity in the other position.

When TKA was first introduced, instrument precision was poor and implantation errors were frequent. There were many pitfalls to overcome, hence the focus was on implant survivorship, rather than reproducing normal knee function (Vendittoli and Blakeney 2017). To simplify and standardize, surgeons introduced the MA technique. This systematic, “one size fits all” approach does not respect the wide range of normal anatomy of the knee (Almaawi et al. 2017). Many studies have illustrated the detrimental effects of soft tissue imbalance on function and long-term survival (Daines and Dennis 2014, Le et al. 2014, Sharkey et al. 2014). It is not well established as to what constitutes the limits of a knee that is considered balanceable with soft-tissue release. Soft tissue balancing is further complicated when attempting to balance both a medial/lateral compartment and flexion/extension space imbalance, with some releases unpredictably affecting both imbalances.

**Figure 4.**
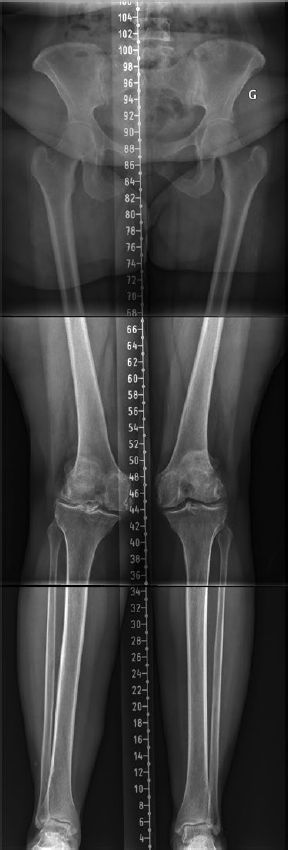
Lower limb long radiographs showing a case with an LDFA of 11° and MPTA of 6°. Reproducing her lower limb alignment with KA technique (unrestricted) would leave her lower limb HKA in 5° of valgus. With rKA, correcting the femur to 5° and the tibia to 2° of varus would results in an HKA of 3° valgus.

With a better understanding of normal knee anatomy and function, KA technique has been introduced to improve clinical results following TKA. KA aims to restore the pre-arthritic patient’s constitutional lower limb alignment and joint surface orientations. It is a joint resurfacing procedure with only exceptional soft tissues release (Howell et al. 2010, Riviere et al. 2017). KA TKA short-term clinical scores and functional evaluation are favorable (Courtney and Lee 2017, Niki et al. 2018, Takahashi et al. 2018, Blakeney et al. 2019b). The implant survivorship at 10 years of a cohort of 220 TKAs by Howell et al. (2018) is very promising. It should be considered, however, that knee anatomy varies widely (Almaawi et al. 2017). Many believe that we should not blindly reproduce all anatomies when performing KA TKA, as some may have deleterious effects on TKA mechanics and clinical outcomes. These extreme anatomies may be inherently mechanically inferior and considered pathological. A strong argument for the existence of patho-anatomies is the unilateral occurrence in some patients. On the other hand, creating a neutral mechanical axis in these patients with MA TKA would generate substantial alteration of the native knee anatomy with subsequent extensive soft-tissue release, severely modifying the physiological joint line orientation and knee mechanics. To address these concerns, rKA has been developed as an alternative solution to the unrestricted KA technique (Hutt et al. 2016, Almaawi et al. 2017) for situations when patients have atypical knee anatomy (Figure 4).

There are still few short- and mid-term follow-up studies on KA TKAs (Howell et al. 2018), whereas MA TKAs have a long history of good survivorship (Font-Rodriguez et al. 1997, Gill et al. 1999, Rodricks et al. 2007). Moreover, the current KA studies include only a limited number of outlier anatomy cases. The rKA is a sound compromise; it reproduces the patient’s constitutional knee anatomy when within a safe range for 50% of cases, requires minor modifications for the rest of the cases, and brings back the extreme anatomies towards acceptable values, modifying their deformities to allow an implant orientation compatible with current materials and fixation methods (Almaawi et al. 2017). As shown in the current study, MPTA and LDFA were modified in the outlier cases by a mean of 0.4° and 1.6° respectively (Table 1). By adhering to the rKA boundaries, it is possible to reliably produce a prosthetic knee with component/knee/limb alignments that always fall within an evidence-based safe alignment range. In a simulation study including 4,884 knees, 17% of knees had very unusual anatomy, with both the femur and tibia articular orientations being in varus or valgus (Almaawi et al. 2017). As both bones contribute the same direction to the overall HKA deviation, using rKA the surgeon needs to decide which bone to correct to fall within the safe range. We believe that the femoral flexion axis plays the more important role in knee kinematics, therefore our practice is to preserve femoral anatomy as closely as possible and perform greater modifications on the tibial side. In our experience, ligamentous releases are usually not needed in cases with anatomic modifications of < 3°. In larger corrections, minimal releases can be added (usually, to a much lesser degree compared with MA) (Hutt et al. 2016). In addition, a study of gait analysis comparing patients operated on with rKA compared with MA technique demonstrated that the rKA patients had knee kinematics that were closer to healthy controls than MA patients (Blakeney et al. 2019b). These kinematic differences translated into a higher postoperative mean KOOS score in the KA group compared with the MA group (74 vs. 61, p = 0.03).

This study has some limitations. The database did not provide demographic data or preoperative diagnosis. We do not know whether any extra-articular deformity was contributing to the alignment. Our results represent only the gaps created by bone resections and do not include additional imbalances linked to physiologic and/or pathologic soft tissue laxity or contracture, or bone loss due to wear. These would impact the final gaps but could not be determined using our method. It is also arguable at what limit a space or compartment imbalance becomes “unbalanceable.” We limited our comparison of the rKA with the MA techniques and did not test the gap balance technique. Finally, we found significant differences for most statistical analyses presented. Our large data set implies a very high analysis power. On the other hand, it is difficult to determine the clinical significance of all measured differences.

In the absence of further evidence from long-term studies of KA TKAs, some authors have cautioned against widespread adoption of the KA technique (Abdel et al. 2014). We believe the rKA protocol offers a satisfactory compromise, allowing re-creation of normal patient anatomy for the majority of cases, avoiding the excessive corrections and ligamentous releases required with MA, but preventing the extremes of implant positioning that a universal KA technique application may produce (Rivière et al. 2019).

### Supplementary data

Data analyzed for varus and valgus native HKA separately can be found in the Supplementary data in the online version of this article, http://dx.doi.org/10.1080/17453674.2019. 1675126

## Supplementary Material

Supplemental Material
