# Supplementary material for: Less gap imbalance with restricted kinematic alignment than with mechanically aligned total knee arthroplasty: simulations on 3-D bone models created from CT-scans
Source: Acta Orthop. 2019 Oct 15;90(6):602–9. doi: 10.1080/17453674.2019.1675126 (PMC6844385; doi:10.1080/17453674.2019.1675126)
Supplement: Supplemental Material [file IORT_A_1675126_SM5740.pdf]

Supplementary data

Table S1. Percentage of knees with medial and lateral gap sizes in extension for MA and rKA techniques in lower limbs with varus or valgus native alignment

	Varus knees						Valgus knees					
Extension	Medial extension gap			Lateral extension gap			Medial extension gap			Lateral extension gap		
space (mm)	MA	rKA	p-value	MA	rKA	p-value	MA	rKA	p-value	MA	rKA	p-value
< 12	23	2.1	< 0.001	6.2	0.0	< 0.001	2.2	0.0	0.01	47	4.1	< 0.001
12–13	41	6.7	< 0.001	33	0.2	< 0.001	11	0.4	< 0.001	42	18	< 0.001
14–15	31	19	< 0.001	41	6.0	< 0.001	44	2.2	< 0.001	10	36	< 0.001
16	4.9	72	< 0.001	21	94	< 0.001	43	97.4	< 0.001	0.4	42	< 0.001

Categorial comparisons with Chi Squared test. The gap size in extension is the sum of the distal femoral bone resection and tibial bone resection. (Note: The sum of the tibial implant thickness and femoral implant thickness is 16 mm).

Table S2. Medial and lateral gap sizes modification in extension and resulting medio-lateral difference in mm for MA and rKA techniques in lower limbs with varus or valgus native alignment. Values are mean (SD) [range]

	Varus knees			Valgus knees		
	MA	rKA	p-value	MA	rKA	p-value
Medial gap	–3.3 (1.8) [–8.9 to 0.0]	–0.6 (1.1) [–6.5 to 0.0]	< 0.001	–1.2 (1.3) [–7.6 to 0.0]	0 (0.3) [–3.8 to 0.0]	< 0.001
Lateral gap	–2.1 (1.5) [8.8 to 16]	–0.1 (0.3) [–2.9 to 0.0]	< 0.001	–4.4 (1.6) [–9.5 to –0.1]	–1.3 (1.5) [–7.1 to 0.0]	< 0.001
ΔML	1.2 (2.4) [–4.7 to 8.9]	0.5 (1.2) [–2.9 to 6.5]	< 0.001	–3.3 (2.0) [–9.5 to 2.1]	–1.3 (1.5) [–7.1 to 1.0]	< 0.001
absolute values	2.0(1.8) [0.0 to 8.9]	0.6 (1.1) [0.0 to 6.5]	< 0.001	3.3 (1.9) [0.0 to 9.5]	1.3 (1.5) [0.0 to 7.1]	< 0.001

The gap size modification is the sum of the distal femoral bone resection and tibial bone resection minus 16 mm (resection goal).
ΔML: lateral gap minus medial gap; a negative value represents a greater medial space than lateral space, whereas a positive value represents a greater lateral than medial space.

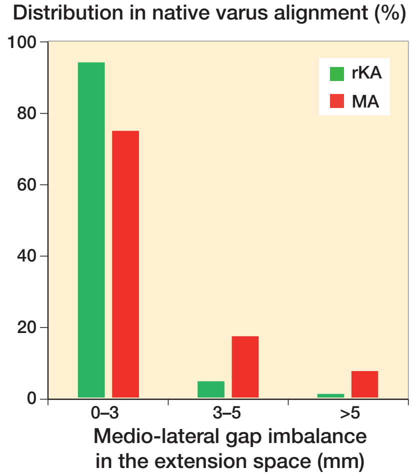

Figure S1. Extension space imbalance (medio-lateral gap difference in mm) for rKA and MA in lower limbs with varus native alignment (p < 0.001).

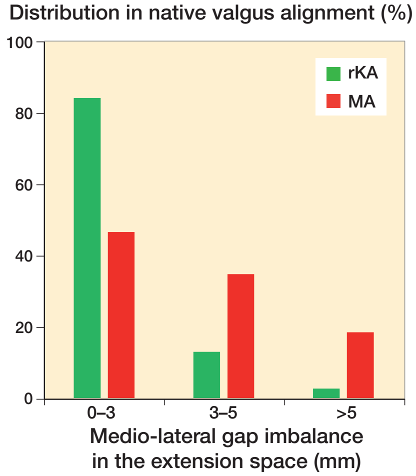

Figure S2. Extension space imbalance (medio-lateral gap difference in mm) for rKA and MA in lower limbs with valgus native alignment (p < 0.001).

Table S3. Medial and lateral gaps sizes modification in flexion at 90° and resulting medio–lateral difference in mm for MA PC method, MA TEA method, and rKA techniques in lower limbs with varus or valgus native alignment. Values are mean (SD) [range]

	MA PC method	MA TEA method	rKA	p-value: rKA vs MA PC	p-value: rKA vs MA TEA
Varus knees					
Medial gap	–1.8 (1.7) [–6.5 to 16]	–0.9 (1.8) [–6.8 to 3.5]	–0.6 (1.1) [–6.5 to 0.0]	< 0.001	< 0.001
Lateral gap	–1.3 (0.3) [–6.5 to –0.9]	–2.3 (0.8) [–7.1 to –0.1]	0.0 (0.2) [–2.0 to 0.0]	< 0.001	< 0.001
∆ML	0.5 (1.8) [–8.1 to 5.3]	–1.4 (2.2) [–9.4 to 5.7]	0.6 (1.2) [–1.8 to 6.5]	0.06	< 0.001
absolute values	1.5 (1.2) [0.0 to 8.1]	2.1 (1.6) [0.0 to 9.4]	0.6 (1.1) [0.0 to 6.5]	< 0.001	< 0.001
Valgus knees					
Medial gap	0.1 (1.3) [–6.5 to 1.7]	1.1 (1.6) [–6.7 to 4.3]	0.0 (0.3) [–3.8 to 0.0]	0.1	< 0.001
Lateral gap	–1.7 (1.0) [–7.3 to –0.9]	–2.7 (1.3) [–7.2 to –0.5]	–0.8 (1.3) [–6.0 to 0.0]	< 0.001	< 0.001
∆ML	–1.8 (2.0) [–8.4 to 5.4]	–3.8 (2.6) [–10 to 5.7]	–0.8 (1.3) [–6.1 to 3.8]	< 0.001	< 0.001
absolute values	2.1 (1.6) [0.0 to 8.4]	3.9 (2.3) [0.0 to 10]	0.9 (1.3) [0.0 to 6.1]	< 0.001	< 0.001

∆ML see Table S2.

Table S4. Flexion-extension differences for the medial and lateral compartments in mm for MA PC method, MA TEA method, and rKA techniques in lower limbs with varus or valgus native alignment. Values are mean (SD) [range]

	MA PC method	MA TEA method	rKA	p-value: rKA vs MA PC	p-value: rKA vs MA TEA
Varus knees					
Medial ∆FE	–1.5 (0.6) [–6.6 to –0.9]	–2.4 (1.0) [–8.2 to –0.3]	0.0 (0.0) [–0.6 to 0.0]	<0.001	<0.001
absolute values	1.5 (0.6) [0.9 to 6.6]	2.4 (1.0) [0.3 to 8.2]	0.0 (0.0) [0.0 to 0.6]	<0.001	<0.001
Lateral ∆FE	–0.7 (1.5) [–5.9 to 1.7]	0.2 (1.8) [–6.1 to 4.4]	0.0 (0.2) [–2.9 to 0.0]	<0.001	<0.001
absolute values	1.4 (1.0) [0.0 to 5.9]	1.5 (1.0) [0.0 to 6.1]	0.0 (0.2) [0.0 to 2.9]	<0.001	<0.001
Valgus knees					
Medial ∆FE	–1.2 (0.1) [–1.7 to –0.9]	–2.2 (0.8) [–4.3 to –0.5]	0.0 (0.0) [0.0 to 0.0]	<0.001	<0.001
absolute values	1.2 (0.1) [0.9 to 1.7]	2.2 (0.8) [0.5 to 4.3]	0.0 (0.0) [0.0 to 0.0]	<0.001	<0.001
Lateral ∆FE	–2.7 (1.5) [–6.5 to 1.2]	–1.7 (1.7) [–6.7 to 2.9]	–0.5 (0.8) [–3.7 to 0.0]	<0.001	<0.001
absolute values	2.8 (1.4) [0.0 to 6.5]	2.0 (1.3) [0.0 to 6.7]	0.5 (0.8) [0.0 to 3.7]	<0.001	<0.001

∆FE: extension gap minus flexion gap; a negative value represents a greater flexion space than extension space, whereas a positive value represents a larger extension than flexion space.

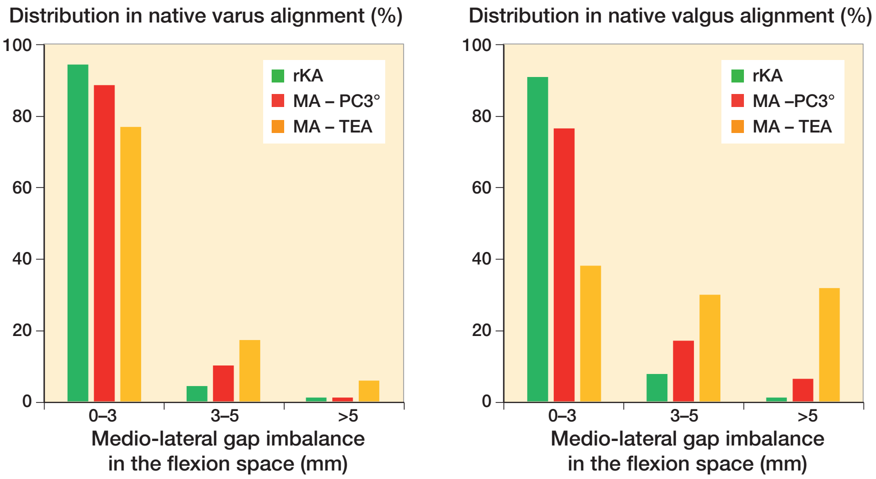

Figure S3. Flexion space imbalance (medio-lateral gap difference in mm) for rKA and MA with PC 3° (p < 0.001) or TEA (p < 0.001) techniques in lower limbs with varus native alignment.

Figure S4. Flexion space imbalance (medio-lateral gap difference in mm) for rKA and MA with PC 3° (p < 0.001) or TEA (p < 0.001) techniques in lower limbs with valgus native alignment.

Table S5. Percentage of knees with medial or lateral compartment imbalance (flexion vs extension gap) for MA PC method, MA TEA method, and rKA techniques in lower limbs with varus or valgus native alignment

	Varus knees						Valgus knees					
	Medial			Lateral			Medial			Lateral		
	MA	rKA	p-value	MA	rKA	p-value	MA	rKA	p-value	MA	rKA	p-value
PC method												
Ext. gap < 15 mm and flex. gap ≥ 16 mm	5.8	0.0	< 0.001	0.0	1.2	0.008	3.8	0.0	< 0.001	0.0	13	< 0.001
Ext. gap ≥ 16 mm and flex. gap < 15 mm	0.0	0.0	N/A	14	0.0	< 0.001	0.0	0.0	N/A	0.0	0.0	N/A
Total	5.8	0.0	< 0.001	14	1.2	< 0.001	3.8	0.0	< 0.001	0.0	13	< 0.001
TEA method												
Ext. gap < 15 mm and flex. gap ≥ 16 mm	23	0.0	< 0.001	0.0	1.2	0.008	24	0.0	< 0.001	0.0	13	< 0.001
Ext. gap ≥ 16 mm and flex. gap < 15 mm	0.0	0.0	N/A	13	0.0	< 0.001	0.0	0.0	N/A	0.0	0.0	N/A
Total	23	0.0	< 0.001	13	1.2	< 0.001	24	0.0	< 0.001	0.0	13	< 0.001

Table S6. Percentage of knees where a space imbalance is present in both the extension and flexion for MA PC method, MA TEA method, and rKA techniques in lower limbs with varus or valgus native alignment

	Varus knees			p-value: rKA vs		Valgus knees			p-value: rKA vs	
	MA PC	MA TEA	rKA	MA PC	MA TEA	MA PC	MA TEA	rKA	MA PC	MA TEA
≤ 3 mm	70	57	94	< 0.001	< 0.001	43	27	84	< 0.001	< 0.001
≤ 5 mm	92	87	99	< 0.001	< 0.001	80	62	97	< 0.001	< 0.001
> 5 mm	0.7	0.5	1.1	0.5	0.2	5.2	13	1.1	0.001	< 0.001
